# Neighborhood Deprivation and Changes in Oral Health in Older Age: A Longitudinal Population-Based Study

**DOI:** 10.1177/00220345231224337

**Published:** 2024-02-27

**Authors:** S.G. Ganbavale, E. Papachristou, J.C. Mathers, A.O. Papacosta, L.T. Lennon, P.H. Whincup, S.G. Wannamethee, S.E. Ramsay

**Affiliations:** 1 Population Health Sciences Institute, Newcastle University, Newcastle upon Tyne, North East England, UK; 2 Department of Primary Care and Population Health, UCL, London, UK; 3 Population Health Research Institute, St George’s, University of London, London, UK

**Keywords:** health inequities, neighborhood characteristics, social deprivation, socioeconomic factors, oral health, aging

## Abstract

The aim of this study was to examine the extent to which neighborhood-level socioeconomic factors (objective and perceived) are associated with poor oral health in older adults over time, independent of individual socioeconomic position. Data for this cross-sectional and longitudinal observation study came from a socially and geographically representative cohort of men aged 71 to 92 y in 2010–12 (*n* = 1,622), drawn from British general practices, which was followed up in 2018–19 (aged 78–98 y; *N* = 667). Dental measures at both times included number of teeth, periodontal pocket depth, self-rated oral health, and dry mouth. Neighborhood deprivation was based on Index of Multiple Deprivation (IMD) and a cumulative index measuring perceptions about local environment. Individual-level socioeconomic position was based on longest-held occupation. Multilevel and multivariate logistic regressions, adjusted for relevant sociodemographic, behavioral, and health-related factors, were performed to examine the relationships of dental measures with IMD and perceived neighborhood quality index, respectively. Cross-sectionally, risks of tooth loss, periodontal pockets, and dry mouth increased from IMD quintiles 1 to 5 (least to most deprived); odds ratios (ORs) for quintile 5 were 2.22 (95% confidence interval [CI], 1.41–3.51), 2.82 (95% CI, 1.72–4.64), and 1.51 (95% CI, 1.08–2.09), respectively, after adjusting for sociodemographic, behavioral, and health-related factors. Risks of increased pocket depth and dry mouth were significantly greater in quintile 5 (highest problems) of perceived neighborhood quality index compared to quintile 1. Over the 8-y follow-up, deterioration of dentition (tooth loss) was significantly higher in the most deprived IMD quintiles after full adjustment (OR for quintile 5 = 2.32; 95% CI, 1.09–4.89). Deterioration of dentition and dry mouth were significantly greater in quintile 5 of perceived neighborhood quality index. Neighborhood-level factors were associated with poor oral health in older age, both cross-sectionally and longitudinally, particularly with tooth loss, and dry mouth, independent of individual-level socioeconomic position.

## Introduction

Socioeconomic conditions of neighborhoods, where individuals are born, reside, and age, significantly influence their health (Solar and Irwin 2010). Neighborhood-level socioeconomic factors can be assessed objectively using census-based measures, such as income, employment, housing, education, and access to services, or subjectively through individuals’ perceptions about neighborhood characteristics, including local services, safety, and environment (Yen et al. 2009). Neighborhood socioeconomic deprivation is linked with health inequalities, whereby individuals residing in socioeconomically deprived neighborhoods have worse health outcomes than those residing in affluent neighborhoods, even after adjusting for individual-level socioeconomic position (Pickett and Pearl 2001). However, the influence of neighborhood environment on oral health remains underexplored (Bower et al. 2007; Turrell et al. 2007; Borrell and Baquero 2011; Steele et al. 2015).

Globally and within the United Kingdom, tooth loss, periodontal (gum) disease, and dry mouth are among the most prevalent health conditions and significantly greater in older age (Petersen and Yamamoto 2005; Gil-Montoya et al. 2015; Ramsay, Whincup, et al. 2015; Bernabe et al. 2020). Oral health problems affect nutritional intake and the well-being of older people, and they are associated with all-cause, cardiovascular, and cancer mortality (Kotronia et al. 2021). Providing dental care is challenging among older adults, who are more likely to suffer from comorbidities (Janto et al. 2022). Consequently, understanding key contributors to oral health in later life is needed.

There is a strong socioeconomic gradient in the burden of poor oral health, with higher rates of oral health problems in socioeconomically disadvantaged individuals (Chavers et al. 2002; Sabbah et al. 2007). Inequalities in oral health are also patterned according to neighborhood-level socioeconomic factors (Bower et al. 2007; Turrell et al. 2007; Borrell and Baquero 2011; Steele et al. 2015). Neighborhood-level factors could be important influences on health in older age, as people in later life are more exposed to their area of residence (Yen et al. 2009). However, current studies are mostly cross-sectional and focus on younger or middle-aged populations (Sanders et al. 2008; Borenstein et al. 2013; Ayo-Yusuf et al. 2016). Therefore, we examined the effects of neighborhood-level deprivation, both cross-sectionally and longitudinally, on a range of subjective and objective oral health measures in a population-based study of older British men. We investigated both objective and subjective measures of neighborhood deprivation. We also investigated whether the influence of neighborhood-level deprivation was independent of individual-level socioeconomic position.

## Materials and Methods

Data for this cross-sectional and longitudinal observation study came from the British Regional Heart Study (BRHS), a cohort study comprising a socially and geographically representative sample of 7,735 British men from 24 towns in Great Britain, initially examined in 1978–1980 (Walker et al. 2004; Lennon et al. 2015). In 2010–2012, surviving participants (*n* = 3,132, age = 71–92 y), were invited to attend a physical examination with an oral health assessment (*n* = 1,722, response rate = 55%), undertaken by 2 trained research nurses, and complete a questionnaire (*n* = 2,137, response rate = 68%) on medical history and behavioral factors at examination or by post. Participants provided written informed consent in accordance with the Declaration of Helsinki. Ethical approval was granted by the National Research Ethics Service (NRES) Committee, London Central region.

The oral health assessment in 2010–2012 comprised a count of natural teeth and 2 measures of periodontal disease assessed in 6 index teeth. Objective measures of periodontal disease included periodontal pocket depth and loss of attachment (Ramsay, Whincup, et al. 2015). Questionnaires included self-reported measures, including self-rated oral health (excellent/good, or fair/poor) (Locker et al. 2005) and dry mouth symptoms (Xerostomia Inventory Scale) (Thomson et al. 1999). Details on assessments and validity have been reported (Ramsay, Whincup, et al. 2015; Ramsay, Papachristou, Watt, Tsakos, et al. 2018). In 2018–2019, surviving participants (*n* = 1,633, age = 78–98 y), attended a follow-up examination, including an oral examination (*n* = 667, response rate = 41%) involving a count of natural teeth and periodontal measures (i.e., periodontal pocket depth, loss of attachment) in all teeth, undertaken by a dental hygienist. A postal questionnaire was completed by 1,009 participants (response rate = 62%). Self-reported measures included self-rated oral health and dry mouth symptoms.

Different oral health measures, including edentulism (no natural teeth), periodontal disease, self-rated oral health, and dry mouth were available. Periodontal disease was defined as periodontal pocket depth of >3.5 mm affecting >20% of the examined sites or loss of attachment of >5.5 mm in more than 20% of the sites examined. Self-rated oral health was grouped into excellent/good versus fair/poor. Dry mouth symptoms were categorized as 0, 1 to 2, and ≥3 symptoms (Ramsay, Papachristou, Watt, Tsakos, et al. 2018).

Neighborhood-level deprivation was measured using the Index of Multiple Deprivation (IMD) for England (McLennan et al. 2011), Scotland (Office of the Chief Statistician and Performance 2012), and Wales (Welsh Assembly Government 2009). These scores are available at lower-layer super output area (LSOA) level, census-based areas (mean population = 1,500 people) in England and Wales, and, for data zones, equivalent areas (mean population = 750 people) in Scotland (Office for National Statistics 2011). IMD is a population-weighted aggregation comprising different “domains” (aspects of deprivation, including income, employment, education, community safety, health, housing, access to services), with a higher score indicating greater deprivation (Ramsay, Morris, et al. 2015). IMD scores for the BRHS cohort were based on LSOAs derived from postcodes of residence in 2010–2012 (Office for National Statistics 2011). The scores were standardized to obtain a composite IMD measure for Great Britain (Abel et al. 2016). IMD scores were divided into quintiles (quintile 1 = least to 5 = most deprived).

In addition, study participants’ perceptions of neighborhood of residence were assessed through questionnaires and included aspects of local area services (social and leisure facilities, health services, transport, etc.), safety, environment (volume of traffic, noise, crime, air quality, etc.), and green spaces. Responses were rated from very poor to very good. Responses to questions on perceptions were averaged to obtain a cumulative index of perceived neighborhood quality (quintile 1 = least to 5 = highest problems).

Covariates included individual social class, smoking, alcohol intake, social interaction, and history of diabetes or cardiovascular disease (CVD), body mass index (BMI), dry mouth medications, and depression. Social class was based on the longest held occupation at entry to the study (age = 40–59 y). Participants were categorized as current smokers, long-term ex-smokers (gave up smoking before 1983), recent ex-smokers, and those who never smoked (Ramsay, Papachristou, Watt, Tsakos, et al. 2018). Alcohol intake was dichotomized as moderate or heavy drinkers (i.e., 5–6 drinks or >6 drinks daily or on most days of the week) and occasional or nondrinkers (Lennon et al. 2015; Kimble et al. 2022). History of diabetes was assessed via self-reported history of diagnosis and/or fasting glucose concentration (>7 mmol/L). History of CVD included self-reported diagnoses of angina, heart attack, or heart failure. Dry mouth medications were based on taking medications with a dry mouth side effect. Social interaction was measured using a social engagement scale comprising 9 social activities (Ramsay et al. 2008). Depression score was measured using 11 items adapted from the Geriatric Depression Scale, a validated self-report measure of the overall life satisfaction and general worries in older adults (Yesavage et al. 1982).

### Statistical Analysis

Multilevel logistic regression models (Wong and Mason 1985) were used to examine the cross-sectional association between IMD quintiles and oral health measures to account for hierarchies in the data comprising individuals nested within areas (LSOAs). IMD quintiles were level 2 variables, whereas age, social class, smoking, alcohol consumption, and history of diabetes or CVD (hereafter, individual-level covariates) were level 1 variables. IMD measures were also fitted as a continuous variable to test for trend across IMD quintiles. The study comprised 1,240 LSOAs with an average of 2 men in each LSOA (810 LSOAs had 1 participant, 209 LSOAs had 2 participants, 103 LSOAs had 3 participants, 103 LSOAs had 4–6 participants, and 15 LSOAs had 7–9 participants). Odds ratios (ORs) with 95% confidence intervals (CIs) were obtained according to quintiles of IMD adjusted for individual. Multivariate logistic regression models were used to obtain age and fully adjusted ORs (95% CIs) for oral health measures according to quintiles of the cumulative perceived neighborhood quality index.

In prospective analyses, the associations of IMD and perceived neighborhood quality index were examined longitudinally with changes in oral health markers from 2010–2012 (baseline) to 2018 (follow-up). Oral health markers included tooth loss/dentition, self-rated oral health, and dry mouth. Changes in oral health were dichotomized as sustained good/improved and sustained poor/deterioration in oral health. Appendix Table 1 details the latter category of each marker.

Multilevel and multivariate logistic regressions were used to calculate ORs with 95% CIs according to quintiles of IMD and perceived neighborhood quality, respectively. We reported 95% CIs to indicate the range of variation of odds ratios, with the width of CIs also indicating the sample size (Yiran et al. 2019). Models were adjusted for individual-level covariates. Changes in periodontal status over time could not be undertaken because these measurements at baseline and follow-up were not comparable (described earlier).

In all models, age was fitted as a continuous variable. The models for dry mouth symptoms were adjusted for dry mouth medications. The models for perceived neighborhood index were additionally adjusted for depression. Social class groups were dichotomized into manual (social classes I, II, III-nonmanual) and nonmanual (III-manual, IV, V). Social class (2 levels), smoking (4 levels), alcohol consumption (2 levels), social interaction (2 levels), and history of CVD or diabetes (2 categories) were fitted as categorical variables. BMI was fitted as a continuous variable. Dry mouth medications were grouped into 0, 1, and 2 or more medications. The lowest quintile of depression scores was defined as no/low level of depression. All analyses were carried out using Stata/SE 14.1, MLwiN 2.32, and SAS 9.4. The study adhered to STROBE (Strengthening the Reporting of Observational Studies in Epidemiology) guidelines (https://www.strobe-statement.org/).

## Results

The physical examination at 71 to 92 y was attended by 1,722 men (response rate = 55%), and questionnaires were completed by 2,137 men (response rate = 68%). Among the 1,722 men who had undergone a dental examination, the prevalence of edentulism (complete tooth loss) was 20%, while 25% had loss of attachment >5.5 mm, and 29% had periodontal pocket depths >3.5 mm. For self-reported oral health outcomes from questionnaires, 34% rated their oral health as fair/poor and 29% had 2 or more dry mouth symptoms. Table 1 presents characteristics of the study population according to IMD quintiles of deprivation. The proportion of men from manual social class groups, prevalence of current smokers, and those with history of CVD or diabetes increased from quintile 1 (least deprived) to quintile 5 (most deprived). Mean BMI was greater in quintiles of higher deprivation. After an 8-y follow-up, 1,009 participants completed the questionnaire, and 667 participants attended an oral health examination in 2018–2019. Data at both time points were available for 935 participants.

**Table 1.** Baseline Characteristics According to Quintiles of Neighborhood-Level Deprivation (Index of Multiple Deprivation) in a Population-Based Study of British Men Aged 71 to 92 years in 2010–2012.

	Neighborhood-Level Deprivation as Measured by Index of Multiple Deprivation					*P* Value for Trend
	Quintile 1 (Least Deprived) (*n* = 605)	Quintile 2 (*n* = 549)	Quintile 3 (*n* = 396)	Quintile 4 (*n* = 318)	Quintile 5 (Most Deprived) (*n* = 263)	
Age, mean ± SD, y	78.76 ± 4.73	78.27 ± 4.62	78.74 ± 4.75	79.12 ± 5.07	79.13 ± 4.91	0.06
Manual occupational social class, *n* (%)	168 (29)	225 (23)	200 (52)	202 (66)	202 (78)	<0.001
Current smokers, *n* (%)	12 (2)	23 (4)	12 (3)	18 (6)	26 (10)	<0.001
History of cardiovascular disease or diabetes, *n* (%)	294 (49)	279 (51)	218 (55)	193 (61)	155 (59)	<0.001
Body mass index, mean ± SD, kg/m^2^	26.40 (3.72)	27.31 (3.77)	27.56 (4.09)	27.57 (3.53)	27.80 (3.99)	<0.001
Moderate or heavy alcohol consumption, *n* (%)	25 (4)	28 (5)	11 (3)	13 (4)	14 (6)	0.85

Table 2 presents ORs (95% CIs) for oral health measures (objective and subjective) from baseline (2010–2012) according to quintiles of neighborhood-level deprivation based on IMD from cross-sectional analyses. The odds of edentulism increased significantly from quintile 1 (least deprived) to quintile 5 (most deprived) (*P* for trend <0.001). Adjustment for social class weakened the associations slightly, but the higher risk in quintiles 4 and 5 remained statistically significant. These increased risks in quintile 4 (OR = 1.87, 95% CI, 1.21–2.87) and quintile 5 (OR = 2.22; 95% CI, 1.41–3.51) remained significant even after adjusting for smoking, alcohol consumption, and history of CVD or diabetes. For periodontal disease measures, the risk of increased pocket depth was associated with IMD quintiles, and the increased risk remained significant (except in quintile 4) with the highest risk being observed in quintile 5 (fully adjusted OR = 2.82; 95% CI, 1.72–4.64). The periodontal disease measure of loss of attachment was not significantly associated with IMD (results not shown). A persistent trend was observed in age-adjusted and fully adjusted models, whereby the risk for edentulism and periodontal disease significantly increased from the least to the most deprived IMD quintile (*P* for trend <0.001). There was an increased risk for fair/poor self-rated oral health for participants in quintile 5 (OR = 1.73; 95% CI, 1.28–2.35); this remained significant upon adjustment for social class but was attenuated on full adjustment. Increased risk of dry mouth among participants in quintiles 2, 4 (borderline), and 5, compared with those in quintile 1, remained after full adjustment (OR for quintile 5 = 1.51; 95% CI, 1.08–2.09).

**Table 2.** Odds Ratios (95% Confidence Intervals) for Objective and Self-Reported Oral Health Measures According to Quintiles of Neighborhood-Level Deprivation (Index of Multiple Deprivation (IMD)) in the British Regional Heart Study Cohort of 2,137 British Men Aged 71 to 92 years in 2010-2012.

IMD Quintile	Objective Oral Health Measures (*n* = 1,722)							
	Edentulism (No Natural Teeth)				Periodontal Disease (20% Sites Affected by Periodontal Pockets >3.5 mm)			
	*n* (%)	Model A^a^	Model B^b^	Model C^c^	*n* (%)	Model A^a^	Model B^b^	Model C^c^
Quintile 1 (least deprived)	63 (13)	1.00	1.00	1.00	84 (21)	1.00	1.00	1.00
Quintile 2	84 (19)	**1.58 (1.10–2.27)**	1.39 (0.96–2.02)	1.29 (0.88–1.91)	102 (30)	**1.54 (1.08–2.19)**	**1.50 (1.05–2.14)**	**1.48 (1.03–2.13)**
Quintile 3	57 (19)	**1.53 (1.03–2.27)**	1.32 (0.87–1.98)	1.17 (0.76–1.81)	74 (32)	**1.74 (1.18–2.55)**	**1.65 (1.11–2.44)**	**1.60 (1.07–2.39)**
Quintile 4	67 (29)	**2.64 (1.78–3.93)**	**2.07 (1.36–3.13)**	**1.87 (1.21–2.87)**	48 (31)	**1.63 (1.06–2.53)**	1.49 (0.95–2.35)	1.39 (0.88–2.21)
Quintile 5 (most deprived)	66 (35)	**3.58 (2.38–5.39)**	**2.60 (1.69–4.01)**	**2.22 (1.41–3.51)**	54 (47)	**3.25 (2.05–5.17)**	**3.09 (1.91–4.99)**	**2.82 (1.72–4.64)**
*P* for trend		<0.001	<0.001	<0.001		<0.001	<0.001	<0.001
IMD Quintile	Self-Reported Oral Health Measures (*n* = 2,137)							
	Fair/Poor Self-Rated Oral Health				Dry Mouth (Xerostomia)			
	*n* (%)	Model A^a^	Model B^b^	Model C^c^	*n* (%)	Model A^a^	Model B^b^	Model C^c,d^
Quintile 1 (least deprived)	192 (33)	1.00	1.00	1.00	231 (39)	1.00	1.00	1.00
Quintile 2	178 (34)	1.05 (0.82–1.35)	1.02 (0.79–1.32)	0.96 (0.74–1.25)	243 (46)	**1.33 (1.05–1.69)**	**1.37 (1.07–1.75)**	**1.35 (1.05–1.73)**
Quintile 3	137 (37)	1.18 (0.90–1.55)	1.09 (0.82–1.44)	1.02 (0.77–1.36)	146 (39)	0.99 (0.76–1.29)	1.02 (0.77–1.34)	1.04 (0.79–1.38)
Quintile 4	95 (32)	0.96 (0.71–1.29)	0.87 (0.64–1.19)	0.81 (0.59–1.12)	136 (46)	1.31 (0.98–1.74)	**1.38 (1.03–1.86)**	**1.35 (1.00–1.83)**
Quintile 5 (most deprived)	114 (46)	**1.73 (1.28–2.35)**	**1.51 (1.10–2.09)**	1.32 (0.94–1.83)	117 (48)	**1.41 (1.04–1.91)**	**1.50 (1.09–2.07)**	**1.51 (1.08–2.09)**
*P* for trend		0.01	0.11	0.48		0.06	0.03	0.03

Bold indicates *P* < 0.05.

a Model A: adjusted for age.

b Model B: adjusted for age and individual social class.

c Model C: adjusted for age, individual social class, smoking status, alcohol consumption, and history of cardiovascular disease or diabetes.

d Model C for dry mouth was additionally adjusted for dry mouth medications.

Table 3 presents ORs (95% CIs) for oral health measures (objective and subjective) according to quintiles of cumulative measure of perceived neighborhood quality. The risk of edentulism increased from quintile 1 (least problems) to quintile 5 (highest problems) (*P* for trend < 0.001). While the risks attenuated after adjusting for social class and other covariates, the risk in quintile 4 remained higher (OR = 1.98; 95% CI, 1.00–3.94). For periodontal disease, increased pocket depth risk was higher in quintiles of greater neighborhood problems in age-adjusted analyses (*P* for trend = 0.02). The increased risk remained significant in quintile 5 (OR = 1.90; 95% CI, 1.00–3.66) upon full adjustment. The odds of fair/poor self-rated oral health increased from quintiles 1 to 5 of perceived neighborhood problems (*P* for trend <0.001); this increased risk was significant in quintiles 4 (OR = 1.58; 95% CI, 1.16–2.14) and 5 (OR = 2.24; 95% CI, 1.64–3.05) in the models adjusted for age and social class. However, these risks attenuated upon full adjustment. The risk of dry mouth was significantly higher in quintiles of greater neighborhood problems in age-adjusted as well as fully adjusted models, with the highest risk in quintile 5 (fully adjusted OR = 2.31; 95% CI, 1.31–4.09).

**Table 3.** Odds Ratios (95% Confidence Intervals) for Objective and Self-Reported Oral Health Measures According to Quintiles of Perceived Neighborhood Quality Index in the British Regional Heart Study Cohort of 2,137 British Men Aged 71 to 92 years in 2010-2012.

Quintile	Objective Oral Health Measures (*n* = 1,722)							
	Edentulism (No Natural Teeth)				Periodontal Disease (20% Sites Affected by Periodontal Pockets >3.5 mm)			
	*n* (%)	Model A^a^	Model B^b^	Model C^c^	*n* (%)	Model A^a^	Model B^b^	Model C^c^
Quintile 1 (least problems)	56 (16)	1.00	1.00	1.00	73 (26)	1.00	1.00	1.00
Quintile 2	45 (13)	0.83 (0.54–1.27)	0.85 (0.54–1.32)	1.06 (0.51–2.19)	79 (30)	1.19 (0.82–1.73)	1.20(0.82–1.75)	1.39 (0.80–2.41)
Quintile 3	76 (23)	**1.52 (1.03–2.24)**	1.43 (0.95–2.13)	1.68 (0.80–3.51)	68 (28)	1.10 (0.74–1.61)	1.05 (0.71–1.55)	1.09 (0.58–2.07)
Quintile 4	59 (19)	1.18 (0.79–1.77)	1.19 (0.78–1.81)	**1.98 (1.00–3.94)**	78 (32)	1.33 (0.91–1.94)	1.31 (0.89–1.92)	1.09 (0.59–2.02)
Quintile 5 (highest problems)	94 (34)	**2.43 (1.66–3.57)**	**2.11 (1.41–3.15)**	1.28 (0.58–2.80)	61 (36)	**1.62 (1.07–2.46)**	1.50 (0.98–2.29)	**1.90 (1.00–3.66)**
*P* for trend		<0.001	<0.001	0.12		0.02	0.07	0.22
IMD Quintile	Self-Reported Oral Health Measures (*n* = 2,137)							
	Fair/Poor Self-Reported Oral Health				Dry Mouth (Xerostomia)			
	*n* (%)	Model A^a^	Model B^b^	Model C^c^	*n* (%)	Model A^a^	Model B^b^	Model C^c,d^
Quintile 1 (least problems)	111 (27)	1.00	1.00	1.00	144 (35)	1.00	1.00	1.00
Quintile 2	127 (32)	1.28 (0.94–1.73)	1.24 (0.91–1.69)	1.30 (0.81–2.10)	136 (35)	0.99 (0.74–1.33)	1.04 (0.78–1.40)	0.94 (0.61–1.45)
Quintile 3	128 (32)	1.28 (0.92–1.73)	1.22 (0.90–1.66)	0.92 (0.52–1.61)	183 (45)	**1.52 (1.15–2.02)**	**1.61 (1.21–2.15)**	1.54 (0.93–2.43)
Quintile 4	149 (38)	**1.63 (1.21–2.20)**	**1.58 (1.16–2.14)**	1.43 (0.86–2.38)	197 (50)	**1.82 (1.37–2.42)**	**1.89 (1.42–2.53)**	1.50 (0.93–2.42)
Quintile 5 (highest problems)	177 (47)	**2.42 (1.80–3.27)**	**2.24 (1.64–3.05)**	1.67 (0.95–2.94)	203 (53)	**2.03 (1.52–2.70)**	**2.21 (1.64–2.98)**	**2.31 (1.31–4.09)**
*P* for trend		<0.001	<0.001	0.09		<0.001	<0.001	0.0007

Bold indicates *P* < 0.05.

a Model A: Adjusted for age.

b Model B: Adjusted for age and individual social class.

c Model C: adjusted for age, individual social class, smoking status, alcohol consumption, history of cardiovascular disease or diabetes, and depression.

d Model C for dry mouth was additionally adjusted for dry mouth medications.

Table 4 presents ORs (95% CIs) from longitudinal analyses for changes in oral health markers according to IMD quintiles. In the age-adjusted model, the risk of experiencing poor/deteriorated dentition increased from quintile 1 to 5; *P* for trend = 0.001. This association slightly weakened but remained significant on adjustment for social class (*P* for trend = 0.007). The increased risks in quintile 3 (OR = 1.74; 95% CI, 1.06–2.83) and quintile 5 (OR = 2.32; 95% CI, 1.09–4.89) remained significant even after full adjustment for smoking, alcohol consumption, history of CVD, or diabetes. The risks of poor/deteriorated self-rated oral health and dry mouth were not significantly associated with IMD quintiles.

**Table 4.** Associations between Quintiles of Neighborhood Deprivation (Index of Multiple Deprivation) and Deterioration of Oral Health over a Period of 8 y in the British Regional Heart Study Cohort of 935 British Men Aged 78 to 98 years.

Quintile	Tooth Loss (*n* = 740)				Self-Rated Oral Health (*n* = 857)				Dry Mouth (Xerostomia) (*n* = 878)			
	*n* (%)	Model A^a^	Model B^b^	Model C^c^	*n* (%)	Model A^a^	Model B^b^	Model C^c^	*n* (%)	Model A^a^	Model B^b^	Model C^c,d^
Quintile 1 (least deprived)	138 (57)	1.00	1.00	1.00	86 (30)	1.00	1.00	1.00	195 (66)	1.00	1.00	1.00
Quintile 2	140 (63)	1.33 (0.91–1.94)	1.23 (0.83–1.81)	1.16 (0.77–1.75)	92 (38)	1.45 (0.96–2.20)	1.39 (0.91–2.11)	1.36 (0.88–2.07)	159 (65)	1.012 (0.66–1.54)	1.00 (0.66–1.53)	–0.04 (–0.47 to 0.38)
Quintile 3	94 (71)	**1.77 (1.12–2.80)**	**1.58 (0.99–2.53)**	**1.74 (1.06–2.83)**	49 (33)	1.27 (0.78–2.06)	1.20 (0.73–1.97)	1.21 (0.74–1.99)	103 (66)	1.26 (0.77–2.07)	1.26 (0.76–2.07)	0.19 (–0.32 to 0.69)
Quintile 4	62 (72)	**1.95 (1.14–3.35)**	1.58 (0.89–2.78)	1.51 (0.83–2.74)	33 (32)	0.94 (0.52–1.71)	0.80 (0.43–1.48)	0.81 (0.44–1.51)	76 (72)	1.46 (0.79–2.69)	1.44 (0.77–2.68)	0.37 (–0.26 to 0.99)
Quintile 5 (most deprived)	47 (80)	**2.93 (1.47–5.85)**	**2.20 (1.08–4.46)**	**2.32 (1.09–4.89)**	25 (36)	1.12 (0.57–2.17)	0.94 (0.48–1.87)	0.96 (0.48–1.91)	54 (72)	1.49 (0.74–2.99)	1.46 (0.72–2.96)	0.31 (–0.41 to 1.05)
*P* for trend		0.001	0.007	0.01		0.98	0.55	0.59		0.09	0.12	0.16

Bold indicates *P* < 0.05

a Model A: adjusted for age.

b Model B: adjusted for age and individual social class.

c Model C: adjusted for age, individual social class, smoking status, alcohol consumption, and history of cardiovascular disease or diabetes.

d Model C for dry mouth was additionally adjusted for dry mouth medications.

Table 5 presents ORs (95% CIs) from longitudinal analyses for changes in oral health markers based on quintiles of the perceived neighborhood quality index. The risk of poor/deteriorated dentition increased from quintile 1 to 5 of perceived neighborhood quality. The increased risks remained significant after full adjustment in quintile 4 (OR = 1.71; 95% CI, 1.01–2.90) and quintile 5 (OR = 1.89; 95% CI, 1.00–3.57) compared with quintile 1. No significant associations were observed for poor/deteriorated self-rated oral health. The risk of poor/deteriorated dry mouth was greater in quintile 4 (OR = 1.69; 95% CI, 1.04–2.77) and quintile 5 (OR = 2.17; 95% CI, 1.24–3.78) than quintile 1, which remained significant after full adjustment.

**Table 5.** Associations between Quintiles of Perceived Neighborhood Quality Index and Deterioration of Oral Health over a Period of 8 years in the British Regional Heart Study Cohort of 935 British Men Aged 78 to 98 years.

Quintile	Tooth Loss (*n* = 745)				Self-Rated Oral Health (*n* = 865)				Dry Mouth (Xerostomia) (*n* = 886)			
	*n* (%)	Model A^a^	Model B^b^	Model C^c^	*n* (%)	Model A^a^	Model B^b^	Model C^c^	*n* (%)	Model A^a^	Model B^b^	Model C^c,d^
Quintile 1 (least problems)	93 (57)	1.00	1.00	1.00	49 (26)	1.00	1.00	1.00	114 (59)	1.00	1.00	1.00
Quintile 2	97 (62)	1.27 (0.81–1.99)	1.24 (0.78– 1.96)	1.36 (0.83– 2.20)	61 (33)	1.50 (0.94– 2.39)	1.49 (0.93–2.39)	1.39 (0.86– 2.24)	110 (59)	0.97 (0.63– 1.48)	0.97 (0.63– 1.49)	1.02 (0.65– 1.59)
Quintile 3	73 (65)	1.38 (0.84– 2.27)	1.17 (0.70– 1.96)	1.42 (0.83– 2.43)	45 (36)	1.52 (0.91– 2.57)	1.45 (0.85– 2.46)	1.38 (0.80– 2.39)	96 (71)	1.60 (0.98– 2.63)	1.56 (0.94–2.58)	1.46 (0.87– 2.44)
Quintile 4	85 (69)	**1.69 (1.03–2.77)**	1.57 (0.94– 2.61)	**1.71 (1.01– 2.90)**	44 (30)	1.21 (0.73– 2.03)	1.11 (0.66– 1.88)	1.09 (0.65– 1.86)	105 (71)	**1.69 (1.04– 2.77)**	**1.70 (1.03– 2.81)**	**1.64 (0.98– 2.73)**
Quintile 5 (highest problems)	75 (77)	**2.39 (1.35– 4.24)**	**1.99 (1.09– 3.62)**	**1.89 (1.00– 3.57)**	42 (38)	1.66 (0.97– 2.84)	1.38 (0.79–2.44)	1.24 (0.69– 2.23)	88 (75)	**2.17 (1.24 3.78)**	**2.21 (1.24– 3.94)**	**2.03 (1.12– 3.68)**
P for trend		0.001	0.01	0.02		0.18	0.54	0.72		0.0005	0.0007	0.002

Bold indicates *P* < 0.05.

a Model A: Adjusted for age.

b Model B: Adjusted for age and individual social class.

c Model C: Adjusted for age, individual social class, smoking status, alcohol consumption, history of cardiovascular disease or diabetes, and depression.

d Model C for dry mouth was additionally adjusted for dry mouth medications.

## Discussion

This study demonstrates a socioeconomic gradient in oral health, particularly in tooth loss and periodontal disease among older adults, according to objective neighborhood-level deprivation, after adjusting for sociodemographic, behavioral, and health-related covariates. Similar gradients are observed in tooth loss and dry mouth according to the perceived neighborhood quality, after adjusting for abovementioned covariates. Tooth loss, periodontal disease, and dry mouth were significantly greater among those in more deprived areas or poorer neighborhood quality, which was independent of individual-level socioeconomic position and other covariates. Longitudinal analyses revealed that residing in neighborhoods with greater deprivation or poorer neighborhood quality was associated with deterioration in oral health, particularly tooth loss and dry mouth.

While these relationships remained significant on adjustment for behaviors (smoking, alcohol consumption), the possibility of residual confounding remains. The increased risk of poorer oral health in older adults in deprived neighborhoods remained after controlling for individual socioeconomic position. This influence of neighborhood-level factors could be driven by variations in access to resources, including housing, healthy foods, healthier environments, social cohesion, and access to services, including health and dental care over the life course (Solar and Irwin 2010; Broomhead 2017; Ramsay, Papachristou, Watt, Lennon, et al. 2018). Older adults living in deprived neighborhoods have lower social capital (Solar and Irwin 2010) and greater exposure to their local neighborhood compared to younger populations (Yen et al. 2009), which could influence their oral health (Broomhead 2017; Ramsay, Papachristou, Watt, Lennon, et al. 2018). Individuals from affluent neighborhoods have better access to knowledge and opportunities to improve and maintain oral health (Solar and Irwin 2010; Broomhead 2017).

The findings of this study align with previous studies demonstrating significant influence of neighborhood-level disadvantage on tooth loss, self-reported oral health, and oral health–related quality of life (Bower et al. 2007; Turrell et al. 2007; Steele et al. 2015; Ayo-Yusuf et al. 2016). Some studies have suggested that mechanisms linking socioeconomic disadvantage with self-rated oral health and periodontal disease operate mainly at a household and individual level (Borrell and Baquero 2011; Borenstein et al. 2013). However, existing studies have mostly focused on middle-aged adults and are mostly cross-sectional (Sanders et al. 2008; Borenstein et al. 2013; Ayo-Yusuf et al. 2016).

The key strength of this study is that it explores the influence of neighborhood-level factors on oral health outcomes in older age in a population-based study comprising socially and geographically representative sample of older men in Great Britain. Both subjective and objective oral health outcomes, which are of particular importance in this older age group, were assessed. While most studies have objectively measured neighborhood deprivation (e.g., IMD), perceived neighborhood quality characteristics, reflecting social capital, have also shown to affect health of older adults (Bowling et al. 2006). Social capital, a key contributor to health outcomes (Solar and Irwin 2010), is not measured within IMD (Department for Levelling Up Housing and Communities 2022). Consequently, perceived neighborhood quality index complemented IMD in capturing various aspects of neighborhood deprivation. Cross-sectional analyses helped explore the associations between neighborhood deprivation and oral health, whereas longitudinal analyses facilitated examining the effects of neighborhood deprivation on changes in oral health over time.

However, the BRHS sample predominantly included White European men. The findings may not be generalizable to women and other ethnicities. Nonattendance of participants with worse general and oral health may have underestimated the influence of neighborhood differences as nonresponders were more likely to be from more deprived areas and with worse health conditions. The lifetime exposure to risk factors, including smoking, is not fully captured and could contribute to residual confounding. Change in deprivation levels over time was not accounted in analyses. Participants may have moved between areas over the follow-up period, but the BRHS has a mostly stable sample at this age. Furthermore, measures of neighborhood-level deprivation could be subject to measurement bias, whereby some individuals living in deprived areas may have high socioeconomic status and vice versa. This bias was mitigated through adjustment for individual socioeconomic status. However, area-level indices measure average distribution of the neighborhood-level factors affecting most of the population living in an area (McLennan et al. 2011). The BRHS data, augmented with IMD quintiles, provided valuable information on objective and subjective measures of oral health and neighborhood circumstances, as well as individual socioeconomic position of older adults collected over time, which are not routinely available within the United Kingdom. Finally, undertaking multiple testing in our analyses could potentially have resulted in false-positive results. Nonetheless, we focused the tests on the primary hypothesis of our study, which is that neighborhood deprivation is associated with poorer oral health in older populations.

This study highlights marked inequalities according to observed and perceived neighborhood deprivation in poor oral health of older adults, particularly in tooth loss, periodontal disease, and dry mouth, independent of individual socioeconomic position. Furthermore, neighborhood deprivation may contribute to deterioration of oral health in older age. Further prospective studies, including women and different ethnic groups, are needed to confirm the generalizability of these findings. Policy and practice initiatives could consider targeted, locally relevant oral health promotion activities and commissioning of dental services using tools such as IMD to promote oral health of older adults in deprived areas.

## Author Contributions

S.G. Ganbavale, contributed to conception, design, data analysis and interpretation, drafted and critically revised the manuscript; E. Papachristou, contributed to conception, design, data analysis, critically revised the manuscript; J.C. Mathers, contributed to conception, design, data interpretation, critically revised the manuscript; A.O. Papacosta, contributed to data acquisition and analysis, critically revised the manuscript; L.T. Lennon, contributed to data acquisition, critically revised the manuscript; P.H. Whincup, S.G. Wannamethee, contributed to conception, design, data acquisition, critically revised the manuscript; S.E. Ramsay, contributed to conception, design, data acquisition, analysis, and interpretation, drafted and critically revised the manuscript. All authors gave final approval and agree to be accountable for all aspects of the work.

## Supplemental Material

sj-docx-1-jdr-10.1177_00220345231224337 – Supplemental material for Neighborhood Deprivation and Changes in Oral Health in Older Age: A Longitudinal Population-Based Study Supplemental material, sj-docx-1-jdr-10.1177_00220345231224337 for Neighborhood Deprivation and Changes in Oral Health in Older Age: A Longitudinal Population-Based Study by S.G. Ganbavale, E. Papachristou, J.C. Mathers, A.O. Papacosta, L.T. Lennon, P.H. Whincup, S.G. Wannamethee and S.E. Ramsay in Journal of Dental Research
