# Supplementary material for: Neighborhood Deprivation and Changes in Oral Health in Older Age: A Longitudinal Population-Based Study
Source: J Dent Res. 2024 Feb 27;103(4):434–41. doi: 10.1177/00220345231224337 (PMC10966931; doi:10.1177/00220345231224337)
Supplement: sj-docx-1-jdr-10.1177_00220345231224337 – Supplemental material for Neighborhood Deprivation and Changes in Oral Health in Older Age: A Longitudinal Population-Based Study [file sj-docx-1-jdr-10.1177_00220345231224337.docx]

**Neighbourhood deprivation and changes in oral health in older age: a longitudinal population-based study**

**SG Ganbavale, E Papachristou, JC Mathers, AO Papacosta, LT Lennon, PH Whincup, SG Wannamethee, SE Ramsay**

**Appendix Table 1: The definitions of different markers of sustained poor/ deterioration in oral health (i.e., dentition/ tooth loss, self-rated oral health, and dry mouth) from baseline (i.e., 2010-12) to follow-up (i.e., 2018-19)**

| Markers of sustained poor/ deterioration in oral health | Definition |
| --- | --- |
| 1. Sustained poor/deteriorated dentition (tooth loss) | 1. Not having a functional dentition (<21 teeth) at both timepoints 2. Change from having a functional dentition (≥21 teeth) at baseline to not having a functional dentition (<21 teeth) 3. Change from having 1-20 teeth to having no teeth at follow-up |
| 1. Sustained poor/deteriorated self-rated oral health | 1. Poor/fair self-rated oral health at baseline and at follow-up 2. Change from good/excellent at baseline to poor/fair self-rated oral health at follow-up |
| 1. Sustained poor/deteriorated dry mouth | 1. Having 1-2 dry mouth symptoms both at baseline and follow-up 2. Change from 1-2 dry mouth symptoms at baseline to ≥3 at follow-up |
